# Ectopic Expression of Rv0023 Mediates Isoniazid/Ethionamide Tolerance via Altering NADH/NAD^+^ Levels in *Mycobacterium smegmatis*

**DOI:** 10.3389/fmicb.2020.00003

**Published:** 2020-02-07

**Authors:** Shailesh Kumar Gupta, Rajendra Kumar Angara, Suhail Yousuf, Chilakala Gangi Reddy, Akash Ranjan

**Affiliations:** ^1^Computational and Functional Genomics Group, Centre for DNA Fingerprinting and Diagnostics, Hyderabad, India; ^2^Graduate Studies, Manipal Academy of Higher Education, Manipal, India; ^3^Regional Centre for Biotechnology, Faridabad, India

**Keywords:** XRE family of protein, *whiB5*, isoniazid resistance, ethionamide resistance, transcription regulation

## Abstract

Tuberculosis (TB) caused by *Mycobacterium tuberculosis* (*Mtb*) accounts for nearly 1.2 million deaths per annum worldwide. Due to the emergence of multidrug-resistant (MDR) *Mtb* strains, TB, a curable and avertable disease, remains one of the leading causes of morbidity and mortality. Isoniazid (INH) is a first-line anti-TB drug while ethionamide (ETH) is used as a second-line anti-TB drug. INH and ETH resistance develop through a network of genes involved in various biosynthetic pathways. In this study, we identified Rv0023, an *Mtb* protein belonging to the xenobiotic response element (XRE) family of transcription regulators, which has a role in generating higher tolerance toward INH and ETH in *Mycobacterium smegmatis* (*Msmeg*). Overexpression of Rv0023 in *Msmeg* leads to the development of INH- and ETH-tolerant strains. The strains expressing Rv0023 have a higher ratio of NADH/NAD^+^, and this physiological event is known to play a crucial role in the development of INH/ETH co-resistance in *Msmeg*. Gene expression analysis of some target genes revealed reduction in the expression of the *ndh* gene, but no direct interaction was observed between Rv0023 and the *ndh* promoter region. *Rv0023* is divergently expressed to *Rv0022c* (*whiB5*) and we observed a direct interaction between the recombinant Rv0023 protein with the upstream region of *Rv0022c*, confirmed using reporter constructs of *Msmeg*. However, we found no indication that this interaction might play a role in the development of INH/ETH drug tolerance.

## Introduction

Tuberculosis (TB) remains a major cause of death worldwide and the leading cause by a single infectious agent (World Health Organisation, 2018). Even though the disease can be cured and managed by several multidrug regimens, the emergence of multidrug-resistant (MDR) TB is proving to be a major challenge for complete eradication of the disease. Worldwide, MDR TB constitutes 3.5% of new TB cases and 18% of previously treated cases (World Health Organisation, 2018). To overcome these challenges and to better counter resistance in *Mycobacterium tuberculosis* (*Mtb*), understanding the mechanisms and deciphering the pathways majorly responsible for generating resistance are greatly required.

Isoniazid (INH), in combination with rifampicin (RIF), ethambutol (EMB), and pyrazinamide (PZA), forms the first-line therapy for TB. Cycloserine, ethionamide (ETH), and amikacin/capreomycin are used as second-line drugs^1^. INH was first used as an anti-TB drug in 1952 and shortly the first INH-resistant *Mtb* (INHr) clinical isolates were reported (Bernstein et al., 1952; Fox, 1952; Middlebrook and Cohn, 1953). INH and ETH are prodrugs that are converted into their active forms by proteins encoded by *katG* (catalase peroxidase KatG) and *ethA* (monooxygenase EthA), respectively (Johnsson and Schultz, 1994; Johnsson et al., 1997; Baulard et al., 2000; DeBarber et al., 2000; Vannelli et al., 2002; Fraaije et al., 2004). Although *katG* and *inhA* (encoding an NADPH-dependent enoyl-ACP reductase) are the main genes involved in INH resistance, clinical isolates with mutations in the *ndh* gene have been identified in INH-resistant *Mtb* strains (Lee et al., 2001). While in the study by Lee et al. (2001), INH resistance mutations were observed only in the *ndh* gene, in other studies, *ndh* mutations occur simultaneously with mutations in other genes (Hazbón et al., 2006; Cardoso et al., 2007). The role of the *ndh* gene mutations in INH and ETH co-resistance in *Mycobacterium smegmatis* (*Msmeg*) and *Mycobacterium bovis* (*Mbovis*) has been shown (Vilcheze et al., 2005), while the role of *ndh* in conferring resistance in *Mtb* is yet to be determined. The gene *ndh* encodes the type II NADH dehydrogenase and its ortholog in *Escherichia coli* (*E. coli*) has been characterized. In *E. coli*, it exists in a monomeric state bound to the membrane, where it oxidizes NADH, reduces quinone, and catalyzes the transfer of electrons from reduced flavin to quinone (Jaworowski et al., 1981; Matsushita et al., 1987; Yagi, 1993; Gennis and Stewart, 1996; Kerscher, 2000). In *Msmeg*, mutations in *ndh* lead to an increase in NADH cellular concentration and inhibition of INH-NAD and ETH-NAD adducts formation (Vilcheze et al., 2005).

Xenobiotic response element (XRE) family of transcription factors are one of the most frequently occurring families of regulators in bacteria. Among the well-studied members of the XRE family are the lambda and Cro repressors, from lambda bacteriophage, and the prophage repressor Xre from *Bacillus subtilis*. The XRE family of regulators share a conserved N-terminal helix-turn-helix (HTH) DNA binding domain, while the C-terminal regulatory region is highly variable. The XRE family of regulators control diverse metabolic functions; e.g., SinR regulates developmental process in *B. subtilis* (Gaur et al., 1991), ClgR regulates *Streptomyces* growth and controls Clp proteolytic complex (Bellier and Mazodier, 2004), PuuR regulates putrescine utilization pathway in *E. coli* K-12 (Nemoto et al., 2012), and BzdR is involved in the anaerobic catabolism of benzoate in the denitrifying *Azoarcus* sp. strain CIB (Barragán et al., 2005). There are seven members of the XRE family of transcription regulators in *Mtb*: Rv0023, Rv0465c, Rv0474, Rv1129, Rv2017, Rv2021, and EspR (*Rv3849*). Except for EspR, which positively regulates the ESX-1 protein secretion system, the principal virulence determinant of *Mtb* (Raghavan et al., 2008), the remaining XRE transcription regulators in *Mtb* are uncharacterized.

Rv0023 is a regulator from the XRE family of transcriptional regulators, known to induce 488 genes and repress 404 genes (Rustad et al., 2014). Rv0023 regulon is enriched for the regulation of NAD reductases (Rustad et al., 2014). *Rv0023* is transcribed in an operon together with *Rv0024*, a gene that codes for an NLPC/p60 family protein and is transcribed divergently from *whiB5*, belonging to the WhiB family of transcriptional regulators. The *whiB5* gene product is a positive regulator of transcription and contributes to *Mtb* virulence and reactivation (Casonato et al., 2012). At present, very little is known about Rv0023 functions and its effect on *Mtb* physiology.

Here, we studied the effects of Rv0023 overexpression in *Msmeg.* The results show that ectopic expression of Rv0023 confers enhanced INH and ETH tolerance in *Msmeg.* Rv0023 ectopic expression downregulates the expression of the *ndh* gene and increases NADH/NAD^+^ levels, which are known to be mediators of INH and ETH resistance in *Msmeg* (Miesel et al., 1998; Vilcheze et al., 2005). We further studied the regulation of the *whiB5-Rv0023* locus and identified Rv0023 as a negative regulator of *whiB5*. We have characterized its binding site and identified the promoters of *Rv0023* and *whiB5*.

## Materials and Methods

### Bacterial Strains and Growth Condition

A complete list of strains used in this study is mentioned in Table 1. Cloning and plasmid propagation were done using *E. coli* strain DH5α. For protein expression, *E. coli* BL21 (DE3) was used. Both strains were grown in Luria Bertani (LB) medium at 37°C. *Mbovis* BCG Pasteur 1173P2, *Msmeg* mc^2^155, and the recombinant strains were grown in Middlebrook 7H9 (Himedia) broth supplemented with 10% OADC (oleic albumin dextrose catalase) (Himedia), 0.2% glycerol, and 0.05% Tween80 (20% stock) or on 7H10 agar without Tween80 at 37°C. Kanamycin (50 μg/ml) (Sigma-Aldrich), ampicillin (100 μg/ml) (Sigma-Aldrich), and hygromycin (50 μg/ml) (Invitrogen) were used as and when required.

**TABLE 1:** List of strains used in this study.

Strain	Chromosomal genotype	Source
*E. coli* DH5α	F– Φ80*lac*ZΔM15 Δ(*lac*ZYA-*arg*F) U169 *rec*A1 *end*A1 *hsd*R17 (rK−, mK+) *pho*A *sup*E44 λ– *thi*-1 *gyr*A96 *rel*A1	Lab repository
*E. coli* BL21 (DE3)	fhuA2 [lon] ompT gal (λ DE3) [dcm] ΔhsdS λ DE3 = λ sBamHIo Δ*Eco*RI-B int:(lacI:PlacUV5:T7 gene1) i21 Δnin5	Lab repository
*M. smegmatis* mc^2^155		Lab repository
*Msmeg*pVV16	*M. smegmatis* mc^2^155 harboring pVV16 plasmid	This study
*Msmeg*pVV0023	*M. smegmatis* mc^2^155 harboring pVV0023 plasmid	This study
*Msmeg*pEJ*whiB5*WT	*M. smegmatis* mc^2^155 harboring pEJwhiB5 plasmid	This study
*Msmeg*pEJ*whiB5*MUT	*M. smegmatis* mc^2^155 harboring pEJwhib5MUT plasmid	This study
*Msmeg*pEJ0023WT	*M. smegmatis* mc^2^155 harboring pEJ0023 plasmid	This study
*Msmeg*pEJ0023MUT	*M. smegmatis* mc^2^155 harboring pEJ0023MUT plasmid	This study
*Msmeg*pEJ*whiB5*-pVV16	*M. smegmatis* mc^2^155 harboring pEJ*whiB5* and pVV16 plasmids	This study
*Msmeg*pEJ*whiB5-*pVV0023	*M. smegmatis* mc^2^155 harboring pEJ*whiB5* and pVV0023 plasmids	This study
*Msmeg*pEJ0023-pVV16	*M. smegmatis* mc^2^155 harboring pEJ*0023* and pVV16 plasmids	This study
*Msmeg*pEJ0023-pVV0023	*M. smegmatis* mc^2^155 harboring pEJ*0023* and pVV0023 plasmids	This study
*Msmeg*pVV0494	*M. smegmatis* mc^2^155 harboring pVV0494 plasmid	Yousuf et al., 2015

### Plasmids and DNA Manipulation

Cloning, genomic, and plasmid DNA isolations were done as per standard molecular biology procedures (Sambrook et al., 1989). The plasmids and primers used in this study are listed in Tables 2, 3, respectively. Overlapping extension PCR was performed to generate site-directed mutations for promoter and critical residues studies. Sequences of all clones generated were confirmed by Sanger sequencing.

**TABLE 2:** List of plasmids used in this study.

Plasmid	Features	Source/reference
pET23a0023	pET21b carrying Rv0023 gene	This study
pEJ414	km^r^ and lacZ reporter vector	Papavinasasundaram et al., 2001
pEJ0023WT	pEJ414 carrying 400bp upstream and 50bp downstream of *Rv0023* start codon	This study
pEJ0023MUT	Derivative of pEJ0023 where TCATAG is mutated to CCAGAG	This study
pEJ*whiB5*WT	pEJ414 carrying 250 bp upstream and 50 bp downstream of *whiB5* start codon	This study
pEJ*whiB5*MUT	Derivative of pEJ*whiB5* where ATACGCTT is mutated to GCACGCGG	This study
pVV16	Hsp60 promoter, Km^r^ and Hyg^r^	Korduláková et al., 2002
pVV0023	pVV16 carrying *Rv0023* gene	This study
pVV0494	pVV16 carrying *Rv0494* gene	Yousuf et al., 2015

**TABLE 3:** List of primers used for cloning.

Clone name	Primer	Sequence	Restriction site
pET0023	0023FP	GGGAATTCCATATGAGCCGTGAGTCGGCCGGCGCGGCC	*Nde*I
	0023RP	CCGCTCGAGCTGCTGCCCCTCATCCGCGTCGTG	Pst1
pEJ0023WT	UP0023FP	CTAGTCTAGAAGCTGTTCGCGCTTTCGGTACTGGC	*Xba*I
	UP0023RP	CCCAAGCTTCGCGAAGTGCGCGAATGGCCGC	*Hin*dIII
pEJ0023MUT	UP0023SDMFP	TGGCCGACCGC**G**CA**GC**GGCGCGTGCCT	*Xba*I
	UP0023SDMRP	AGGCACGCGCC**GC**TG**C**GCGGTCGGCCA	*Hin*dIII
pEJ*whiB5*WT	UP*whiB5*FP	CTAGTCTAGAGAGCTGTGCTTCGGCGTAGC	*Xba*I
	UP*whiB5*RP	CCCAAGCTTATCGGGGTACCCGAACC	*Hin*dIII
pEJ*whiB5*MUT	UP*whiB5*SDMFP	CACAGACAT**GC**ACGC**GG**TTGCCTATGTTTCGTTCAACAA	*Xba*I
		GGAGGCCGGCACAAGCTTGGG	
	UP*whiB5*SDMRP	CCCAAGCTTGTGCCGGCCTCCTTGTTGAACGAAACATAGG	*Hin*dIII
		CAA**CC**GCGT**GC**ATGTCTGTG	
pVV0023	pvv0023FP	GGGAATTCCATATGAGCCGTGAGTCGGCCGGCGCGGCC	*Nde*I
	pvv0023RP	AAAACTGCAGCTGCTGCCCCTCATCCGCGTCGTG	*Hin*dIII
pEJUF1	UF1FP	GGGCCTGAGCTATCTGGAGCGCG	
	UF1RP	CGCGCTCCAGATAGCTCAGGCCC	
pEJUF2	UF2FP	CTGTGCTTTTGTGTGGCTTGCG	
	UF2RP	CGCAAGCCACACAAAAGCACAG	

*Restriction site in the primer was underlined. The point mutations incorporated in SDM primers were highlighted in bold.*

### Protein Expression and Purification

pET23a-0023 plasmid containing *Rv0023* ORF was used to transform *E. coli* BL21 (DE3) strain. Cells were grown in LB broth at 37°C till mid-log phase, and then 1 mM IPTG (isopropyl 1-thio-β-D-galactopyranoside) was added to induce the expression of protein. Cells were grown for another 4 h at 37°C, after which cells were harvested and purified using Ni-NTA affinity chromatography as described earlier (Yousuf et al., 2018). The purity of recombinant protein was analyzed by 12% SDS-PAGE and protein concentration was measured by Bradford assay.

### β-Galactosidase Assay

*Mycobacterium smegmatis* mc^2^155 strain was transformed with various constructs as required and grown in 7H9 media. The cultures were grown to mid-log phase and β-galactosidase activity was measured in Miller Units (MU) and all experiments were done in triplicate (Miller, 1972).

### Electrophoretic Mobility Shift Assay

To verify the interaction between Rv0023 and the upstream region of *whiB5*, electrophoretic mobility shift assay (EMSA) was performed. The 500-bp upstream region and the 50-bp downstream region of *whiB5* were PCR amplified and labeled with γ-^32^P ATP (3000 Ci mmol^–1^) using T4 polynucleotide kinase as per manufacturer’s instructions (New England Biolabs). Labeled DNA was purified using nucleotide purification kit (Qiagen). Purified labeled DNA was then incubated with increasing concentrations of recombinant Rv0023 protein in an EMSA reaction buffer (25 mM HEPES, pH 7.9, 0.1 mM EDTA, 10 mM MgCl_2_, 20 mM KCl, and 5% glycerol) with 50 ng/μl poly(dI–dC) as a non-specific DNA competitor. The protein–DNA complex was incubated for 45 min and was resolved on a 6% non-denaturing polyacrylamide gel in 0.5 × tris-borate (TBE) buffer. The gel was run for 3–4 h to allow sufficient resolution of protein–DNA complex.

Electrophoretic mobility shift assay with commercially synthesized overlapping oligonucleotide and motifs was performed to identify the exact binding region of Rv0023.

### Primer Extension

To map the (+1) transcription start site (TSS), primer extension was performed as described earlier and in Cold Spring Harbor protocols (Carey et al., 2013; Angara et al., 2018). As the ORFs and intergenic region of *whiB5-Rv0023* locus are 100% conserved between *Mtb* and *Mbovis*, we used *Mbovis* RNA for primer extension studies. RNA was isolated with the Qiagen RNeasy kit as per manufacturer’s instructions. SuperScript III reverse transcriptase (Invitrogen) was used to transcribe cDNA from total RNA (10–15 μg) using 5′-end labeled γ-^32*P*^ ATP primers (Table 4). Primer extension products were resolved on 6% polyacrylamide/8 M urea gel in TBE buffer. The size of primer extension product was determined by generating a dideoxy sequencing ladder using pUC19 plasmid as template and M13 universal primer (USB; 70140 KT).

**TABLE 4:** List of primers used in primer extension.

Primer	Sequence
0023PE1	CTCACGGCTCACCGCACGCTCCG
0023PE2	CGCGAAGTGCGCGAATGGCCGC

### Stress Assay

To check the sensitivity of *Msmeg* toward various stress conditions, *Msmeg*WT, *Msmeg*pVV16, and *Msmeg*pVV0023 cells were grown in complete 7H9 media (supplemented with 10% OADC, 0.2% glycerol, and 0.05% Tween80) at 37°C overnight. Then, the cells were centrifuged and washed with PBS. The cells were then suspended in 7H9 media and the cell concentration was adjusted to 0.02 OD at 600 nm. The sensitivity of bacterial cultures toward 0.1% SDS (Sigma Aldrich) and 5 mM hydrogen peroxide (Merck) was measured at 37°C for 6 h. Sensitivity to 250 μg/ml lysozyme (Sigma Aldrich) was measured at 37°C for 24 h. Susceptibility of *Msmeg* toward the antibiotics (Sigma Aldrich) INH (10 μg/ml, MIC of 5 μg/ml), RIF (10 μg/ml, MIC of 1 μg/ml), and ETH (2.5 μg/ml, MIC of 10 μg/ml) was determined at 37°C for 24 h. For determining CFU numbers for each stress condition and antibiotics, the cells were serially diluted (10-fold) and plated onto 7H10 agar plates.

Further, the strains *Msmeg*WT, *Msmeg*pVV16, and *Msmeg*pVV0023 were serially diluted (10-fold) and spotted on the 7H10 agar plates containing increasing concentrations of INH (0, 5, 10, and 15 μg/ml) and ETH (0, 2.5, and 5 μg/ml). The plates were incubated at 37°C for 48 h.

### qRT-PCR

Total RNA from *Msmeg*pVV16 (vector control) and *Msmeg*pVV0023 (Rv0023 protein overexpressed) cultures were isolated using Qiagen RNeasy kit. SuperScript III reverse transcriptase (Invitrogen) was used to generate cDNA using random hexamers. Real-time PCR was carried on the BioRad CFX96 system using gene-specific primers (Table 5) and EvaGreen qPCR mastermix (Applied Biological Materials Inc.) as per standard protocol. The fold change in expression relative to *Msmeg*pVV16 (vector control) was calculated after normalizing to *sigA*. The 2^−ΔΔ*CT*^ method was used to calculate relative changes in gene expression (Livak and Schmittgen, 2001).

**TABLE 5:** List of primers used in real-time PCR.

Primer	Sequence
inhARTFP	GTGAGGGCAACAAGATCGAC
inhARTRP	GTACGAGTACGCCGAGATGT
*ndh*RTFP	CGGGTTCAAGACGAAGATCG
*ndh*RTRP	CTTTCTCGGTGTCCTGCAC
katGFP	CCAAGTGGGACAACAGCTTC
katGRP	GAGATCCGAGGTCAGCATCG
ethAFP	CGCCGAGAAGACCAACAAAT
ethARP	AATGCTTCTGCACGTCGAAA

### NADH/NAD^+^ Cellular Concentration

To measure the cellular concentration of NADH and NAD^+^, *Msmeg*pVV16 and *Msmeg*pVV0023 cells were grown to an OD_600_ of 0.8–1.2. Cells were collected (1 ml) by centrifugation (11,000 rpm for 2 min). The supernatant was removed and 300 μl of 0.2 M HCl (for NAD^+^ extraction) or 0.2 M NaOH (for NADH extraction) was added to the cells. The cells were resuspended and incubated at 50°C for 10 min after which extracts were cooled to 0°C. The bacterial suspensions were neutralized by adding 0.1 M HCl (for NADH extraction) or 0.1 M NaOH (for NAD^+^ extraction) dropwise while vortexing at high speed. Centrifugation was done to remove the cell debris and supernatant was transferred to a new tube and used immediately. NADH and NAD^+^ concentrations were obtained by spectrophotometrically measuring the rate of MTT [3-(4,5-dimethylthiazol-2-yl)-2,5-diphenyl tetrazolium bromide, 4.2 mM] (Amresco), reduction by yeast alcohol dehydrogenase II (Sigma Aldrich) in the presence of PES (phenazine ethosulfate, 16.6 mM) (Sigma Aldrich) at 570 nm (Leonardo et al., 1996; San et al., 2002; Vilcheze et al., 2005). The concentration of nucleotide (NADH/NAD^+^) is proportional to the rate of MTT reduction.

### Bioinformatics

Rv0023 protein sequence (UniProt P9WMI3) was used as a query in the SynTax web server^2^ for synteny analysis within the *Mycobacteriaceae* family (Oberto, 2013); a conserved domain database search was performed to identify HTH_3 and XRE domains (Marchler-Bauer et al., 2015). Orthologous sequences of the *whiB5–Rv0023* intergenic region from different mycobacterial species were retrieved from KEGG genome database and aligned using CLUSTAL OMEGA (Sievers et al., 2011).

### Statistical Analysis

Data were presented as mean ± SD. Student’s *t-*test and one-way ANOVA were used to determine the statistical significance between groups and values with *p* < 0.05 were considered to be significant. GraphPad Prism software version 5.02 was used for the statistical analysis.

## Results

### Overexpression of Rv0023 Confers Increased INH and ETH Tolerance in *Msmeg*

**FIGURE 1:**
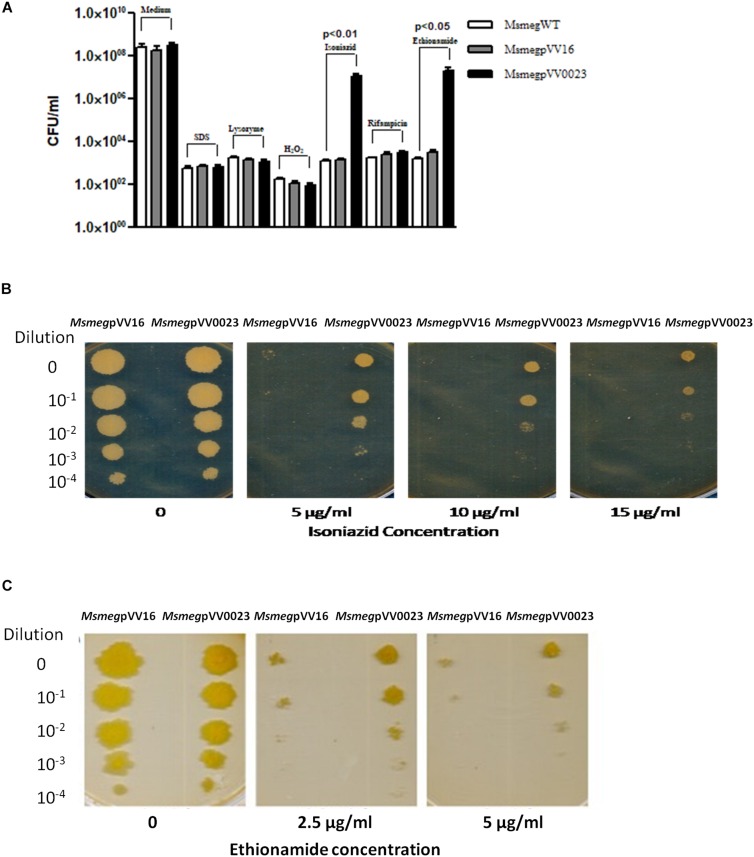
Survivability of Rv0023 expressing *Msmeg* in stress conditions. **(A)** The *Msmeg*WT, *Msmeg*pVV16, and *Msmeg*pVV0023 were subjected to 0.1% SDS for 6 h, 250 μg of lysozyme for 24 h, 5 mM hydrogen peroxide for 6 h, INH (10 μg/ml), RIF (10 μg/ml), and ETH (5 μg/ml) for 24 h. Cells were plated on 7H10 agar plates and bacterial CFU were counted. Data are presented as the mean ± SD from three biological replicates. **(B)** *Msmeg*pVV16 and *Msmeg*pVV0023 cells were serially diluted and spotted on plates containing increasing concentrations of INH. **(C)** *Msmeg*pVV16 and *Msmeg*pVV0023 cells were serially diluted and spotted on plates containing increasing concentrations of ETH.

*Rv0023* is a non-essential gene in *Mtb*, yet it regulates a high number of genes in the genome (Sassetti et al., 2003; Rustad et al., 2014). *Msmeg* genome does not harbor the ortholog of Rv0023 and hence serves as a good model to study the function of Rv0023. To ascertain the role of *Rv0023*, we constructed an overexpressing strain of Rv0023 in *Msmeg*, *Msmeg*pVV0023. The wild-type *Msmeg*WT, vector control *Msmeg*pVV16, and *Msmeg*pVV0023 strains were subjected to acid fast staining to rule out the effect of vector insertion on cellular integrity. The cells were found to be intact and acid fast (Supplementary Figure 1). To further study the role of Rv0023 in cellular physiology, *Msmeg*WT, *Msmeg*pVV16, and *Msmeg*pVV0023 were subjected to different stress conditions as depicted in Figure 1A, and tolerance was measured in terms of CFU. The CFU was counted for all conditions and we observed that overexpression of Rv0023 leads to increased tolerance with regard to INH and ETH, and no effect was observed with any other stress conditions. Tolerance to INH and ETH was not seen either on the wild type or in vector control strains (Figure 1A).

The role of Rv0023 in generating tolerant strains was further confirmed via spotting the wild type, vector control, and Rv0023 overexpressed strains in the presence of increasing concentrations of INH and ETH. *Msmeg*WT, *Msmeg*pVV16, and *Msmeg*pVV0023 cells were serially diluted and spotted on plates containing 5, 10, and 15 μg/ml of INH and 2.5 and 5 μg/ml of ETH. Even at higher concentrations, *Msmeg*pVV0023 showed tolerance toward INH and ETH (Figures 1B,C and Supplementary Figure 2). The results indicate that Rv0023 expression specifically contributes toward the higher tolerance of INH and ETH in *Msmeg.*

### Rv0023 Expression in *Msmeg* Alters the NADH/NAD^+^ Levels

**FIGURE 2:**
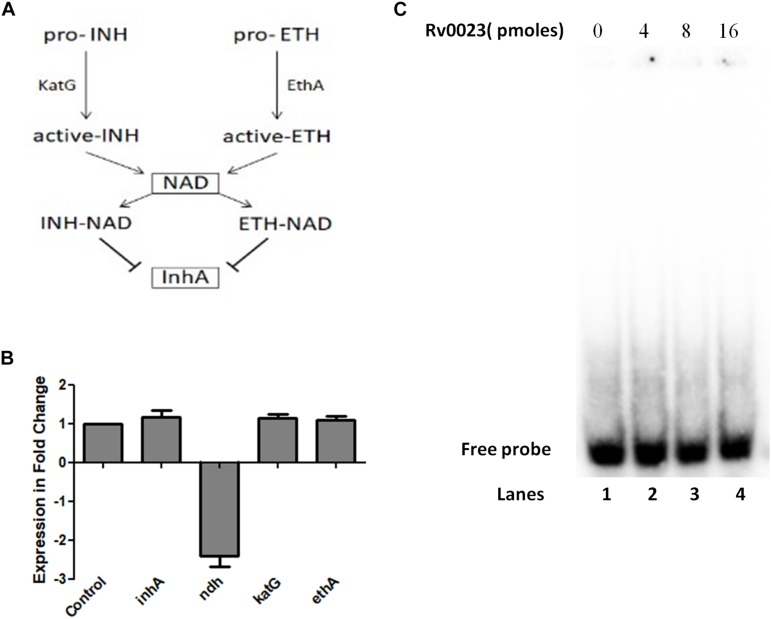
*ndh* gene regulation by Rv0023. **(A)** Schematic representation of mechanisms of action of INH and ETH. **(B)** Expression levels of genes involved in INH and ETH resistance pathway in *Msmeg*pVV16 and *Msmeg*pVV0023 cells. Fold change was calculated relative to the vector control pVV16. Data are presented as the mean ± SD from three biological replicates. One-way ANOVA was applied and the *p-*value obtained was *p* < 0.0001. **(C)** EMSA studies with radiolabeled 300 bp of upstream region of *ndh*. Lane 1: probe without any protein. Lanes 2–4: probe with increasing concentration of purified Rv0023 protein.

Isoniazid and ethionamide are both prodrugs and they are activated by the protein catalase–peroxidase KatG and the NADPH-specific flavin adenine dinucleotide-containing monooxygenase, EthA, respectively. After activation, they react with NAD^+^ to form INH-NAD and ETH-NAD adduct. This species then inhibits the enoyl ACP reductase InhA, which leads to the inhibition of mycolic acid biosynthesis and eventual mycobacterial cell death (Figure 2A). The major common genes to both pathways are the *ndh* (maintains NADH/NAD^+^ ratio) and *inhA*. *Msmeg* orthologs of these and other important genes involved in INH and ETH resistance were subjected to qRT-PCR quantification. Total RNA was isolated from *Msmeg*pVV16 and *Msmeg*pVV0023 and the expression levels compared between the two strains. We found that the expression level of *ndh* (MSMEG_3621) in *Msmeg*pVV0023 was approximately twofold lower than that of *Msmeg*pVV16. No changes in the expression levels were observed in *inhA* (MSMEG_3151), *katG* (MSMEG_6384), and *ethA* (MSMEG_6440) between both strains (Figure 2B). As *ndh* encodes NdhII, which oxidizes NADH to NAD^+^, we reasoned that the ratio of NADH/NAD^+^ might be altered in *Msmeg*pVV0023 strain. So, we measured the cellular ratio of NADH and NAD^+^ in *Msmeg*pVV16 and *Msmeg*pVV0023 strains and found that the ratio of NADH/NAD^+^ is increased in *Msmeg*pVV0023

(Table 6). These results indicate that Rv0023 expression alters both the transcript levels of *ndh* gene and NADH/NAD^+^ levels. To find out whether these effects are directly mediated by Rv0023 or through some indirect means, we studied the interaction of recombinant Rv0023 with the upstream region of *ndh* (MSMEG_3261) gene. However, under the experimental conditions mentioned, we did not observe any such positive interaction (Figure 2C).

**TABLE 6:** Cellular concentration of NADH and NAD^+^.

Strain	NADH (μM)	NAD^+^ (μM)	NADH/NAD^+^
MsmegpVV16	8.29	9.93	0.83
*Msmeg*pVV0023	11.27	9.44	1.19

### Rv0023 Negatively Regulates *whiB5*

**FIGURE 3:**
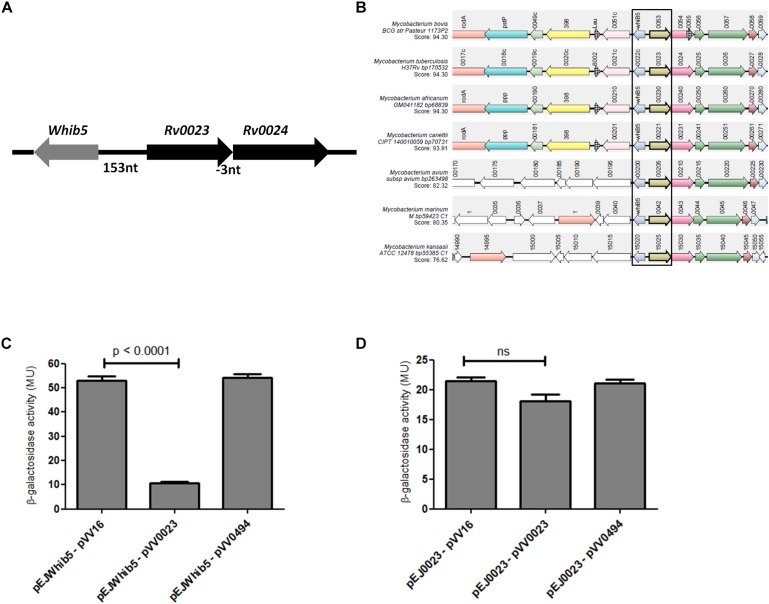
Rv0023 exists in a syntenic relationship with *whiB5* and negatively regulates *whiB5* expression. **(A)** Genomic organization of *whiB5* and *Rv0023* locus. **(B)** Schematic representation showing synteny of *Rv0023* and *whiB5* (within rectangular boxes) from seven mycobacterial species strains. **(C)** β-Galactosidase activity of *whiB5* promoter with ectopic expression of Rv0023 protein and Rv0494 (non-specific protein for negative control). **(D)** β-Galactosidase activity of *Rv0023* promoter in the presence of Rv0023 and Rv0494 overexpression.

*Rv0023* is in operon with *Rv0024* and is transcribed divergently from *whiB5* (Figure 3A). *Rv0023* and *whiB5* are absent from the non-pathogenic, fast-growing *Msmeg* but are present in *Mtb*. So, we tried to find the co-occurrence of these two genes in other mycobacterial species. Synteny analysis of *Rv0023* and *whiB5* was performed for important species of the *Mycobacterium* genus using the “SynTax” web server (Figure 3B). It was observed that *whiB5* is in synteny with *Rv0023* and that both are present only in pathogenic species of mycobacteria (Supplementary Table 1).

The syntenic relationship of the two regulators prompted us to probe the regulation of the *whiB5-Rv0023* locus. To identify the regulatory elements at this locus, upstream regions of *whiB5* and *Rv0023* were cloned in the promoter-less vector pEJ414 and named pEJ*whiB5* and pEJ0023, respectively. The coding region of *Rv0023* was cloned in the pVV16 vector to obtain pVV0023. *Msmeg* was transformed with pEJ*whiB5* or pEJ0023. The strains were again transformed with pVV023 to obtain *Msmeg*pEJ*whiB5*-pVV0023 and *Msmeg*pEJ0023-pVV0023 double transformants. Promoter activities were measured in double transformants (*Msmeg*pEJ*whiB5*-pVV0023, *Msmeg*pEJ*whiB5*-pVV16, *Msmeg*pEJ0023-pVV0023, and *MsmegpEJ0023*-pVV16). Overexpression of Rv0023 caused approximately fivefold decrease in *whiB5* promoter activity (Figure 3C), whereas no significant change in the promoter activity of *Rv0023* was noted (Figure 3D). Rv0494, a FadR transcriptional regulator, was used as a negative control, which did not affect the promoter activities of any of these genes. These data suggest that Rv0023 negatively regulates the expression of *whiB5*, but it is not auto-regulatory.

### Rv0023 Binds to *whiB5 in vitro*

**FIGURE 4:**
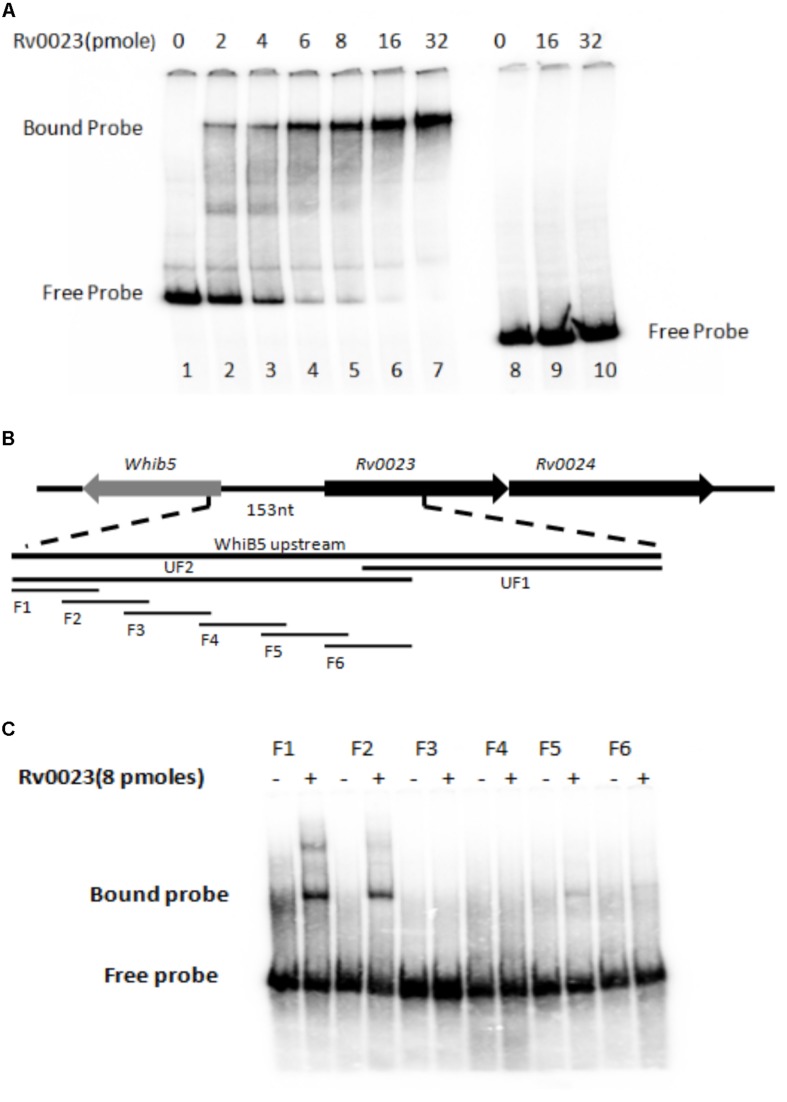
Rv0023 interaction with *whiB5* upstream. **(A)** Rv0023 binding to *whiB5* upstream in EMSA. Lane 1: radiolabeled *whiB5* upstream with no protein. Lanes 2–7: *whiB5* upstream with increasing concentration of Rv0023 protein. Lane 8: upstream region of Rv0494, a non-specific DNA, with no protein. Lanes 9–10: upstream region of Rv0494, a non-specific DNA, with increasing concentration of Rv0023 protein. **(B)** Six overlapping fragments (F1–F6) covering *whiB5* and *Rv0023* locus. **(C)** EMSA for overlapping fragments of *whiB5* upstream with purified Rv0023 protein. Lanes F1–F6: fragments with (+) and without (–) Rv0023 (8 pmol) protein.

As we have observed that Rv0023 expression represses the promoter activity of *whiB5* in *Msmeg*, it prompted us to further test whether Rv0023 interacts with *whiB5* promoter or not. Five hundred base pairs upstream and 50 bp downstream of *whiB5* gene containing the promoter were radiolabeled and EMSA was performed with the recombinant Rv0023. The radiolabeled upstream region of *whiB5* interacted with the purified recombinant Rv0023 in a dose-dependent manner. No interaction of Rv0023 was observed with non-specific DNA (Figure 4A).

**FIGURE 5:**
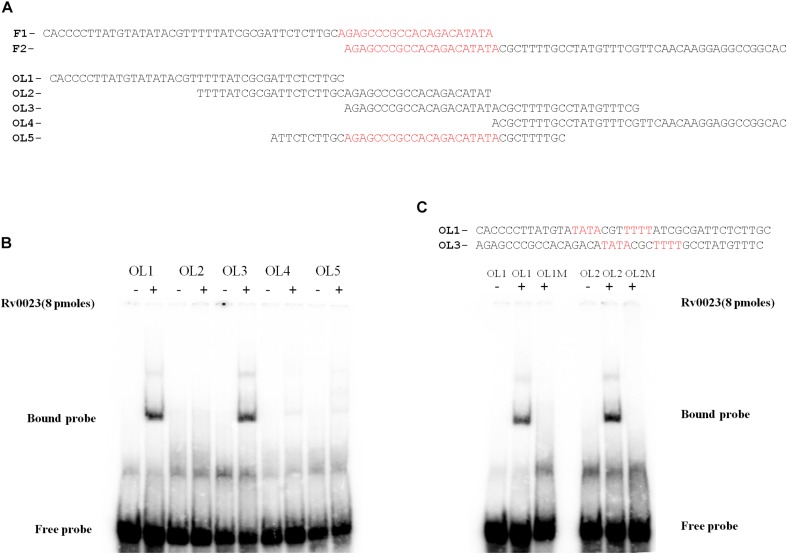
Identification of Rv0023 binding site. **(A)** OL1–OL4 spanning the F1–F2 fragments and OL5 comprising the overlapping region of F1–F2. **(B)** EMSA with fragments OL1–OL5 with (+) and without (–) Rv0023 (8 pmol) protein. **(C)** OL1 and OL3 fragments containing the imperfect palindrome, indicated in red. EMSA with mutated imperfect palindromic DNA region. Fragments with (+) and without (–) Rv0023 (8 pmol) protein.

Both EMSA and β-galactosidase assays confirmed that Rv0023 interacts with *whiB5* upstream and negatively regulates *whiB5* expression. In order to find the exact binding site of Rv0023, the 550 bp upstream of *whiB5* was divided into two fragments: UF1 of 340 bp and UF2 of 250 bp (Figure 4B). We observed that only the UF2 fragment binds to Rv0023 (Supplementary Figure 3). The UF2 fragment was further divided into six overlapping fragments, F1–F6 (Figure 4B and Table 7). Purified Rv0023 binds to fragments F1 and F2, whereas no binding was observed with other fragments (Figure 4C). So, we looked for a binding site at the overlapping region of F1 and F2. We generated new oligonucleotides (OL1–OL5) covering the F1–F2 overlapping region (Figure 5A and Table 8). EMSA with these radiolabeled fragments showed that Rv0023 binds to OL1 and OL3 fragments whereas no binding was observed with other fragments (Figure 5B). Close examination of these fragments revealed the presence of an imperfect palindrome present in both sequences, TATAcgtTTTT in OL1 and TATAcgcTTTT in OL3 (Figure 5C). To confirm the binding sites identified, we mutated TATAcgtTTTT to TGTGcgtTGTG in OL1 and TATAcgcTTTT to TGTGcgcTGTG in OL3 to obtain OL1M and OL3M, respectively (Table 8). Binding studies have shown that Rv0023 binds to the native OL1 and OL3 fragments, but not to the mutant fragments, OL1M and OL3M (Figure 5C). We conclude that there are two binding sites (TATAcgtTTTT and TATAcgcTTTT) of Rv0023 in the upstream region of *whiB5* comprising an 11-bp imperfect palindromic sequence with a 3-bp spacer region.

**TABLE 7:** List of overlapping fragments used in Figure 4.

Overlapping fragment	Sequence
F1	AGAGCCCGCCACAGACATATACGCTTTTGC CTATGTTTCGTTCAACAAGGAGGCCGGCAC
F2	CACCCCTTATGTATATACGTTTTTATCGCG ATTCTCTTGCAGAGCCCGCCACAGACATAT
F3	CCATAAGCCGGATACTACCCGGATACGACT CGGCCGCGGCCACCCCTTATGTATATACGT
F4	TGGCCGCGCCGGCCGACTCACGGCTCACCG CACGCTCCGGCCATAAGCCGGATACTACCC
F5	TCCGCGAGGGACCAGTCACGCGACTCGCGA AGTGCGCGAATGGCCGCGCCGGCCGACTCA
F6	GCTCAGGCCCATGGTGCTTACGCCAGTGGC GGCCGCCAGGTCCGCGAGGGACCAGTCACG

**TABLE 8:** List of overlapping fragments used in Figure 5.

Overlapping fragment	Sequence
OL1	CACCCCTTATGTATATACGTTTTTATCGCGATTCTCTTGC
OL2	TTTTATCGCGATTCTCTTGCAGAGCCCGCCACAGACATAT
OL3	AGAGCCCGCCACAGACATATACGCTTTTGCCTATGTTTCG
OL4	ACGCTTTTGCCTATGTTTCGTTCAACAAGGAGGCCGGCAC
OL5	ATTCTCTTGCAGAGCCCGCCACAGACATATACGCTTTTGC
OL1M	CACCCCTTATGTAGAGACGTGTGTATCGCGATTCTCTTGC
OL3M	AGAGCCCGCCACAGACAGAGACGCGTGTGCCTATGTTTCG

### Analysis of Rv0023 Binding Site

**FIGURE 6:**
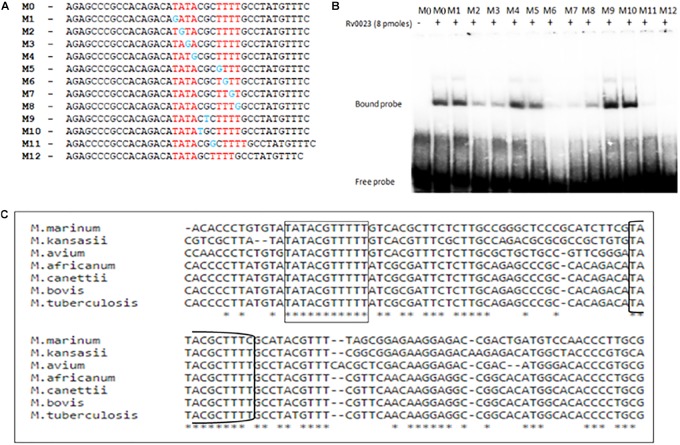
Identification of important residues in the imperfect palindrome for Rv0023 binding. **(A)** Representation of probes (∼40 bp each) containing native binding site M0 and probe with mutation at specific positions in the binding site M1–M8 and probe with mutation in spacer regions M9–M12. **(B)** EMSA with radiolabeled probes [native (M0), mutated (M1–M9), and altered (M9–M12)] with constant concentration of purified Rv0023 protein. Fragments with (+) and without (–) Rv0023 (8 pmol) protein. **(C)** Multiple sequence alignment of *whiB5* upstream from selected members of mycobacterial species. The two binding sites of Rv0023 are highlighted in rectangular boxes.

The importance of each residue within the Rv0023 binding site was determined by generating 39 bp probes (M0–M12) containing specific point mutations on each half of the imperfect palindrome and in the spacer region (Figure 6A). The probes were radiolabeled and were used in binding studies with recombinant Rv0023. The binding was observed with probes M0 (unaltered), M1, M4, and M5, and significant loss of binding was observed with M2, M3, M6, M7, and M8 fragments, suggesting the importance of these residues in Rv0023 binding. Changing the residues in the spacer region (M9–M10) did not affect Rv0023 binding; however, changing the length of spacer region by one base lead to significant loss of Rv0023 binding (M11–M12) (Figure 6B). As mentioned earlier, the organization of *Rv0023* and *whiB5* is conserved across pathogenic mycobacterial species. Therefore, we looked into the conservation of identified Rv0023 binding sites across the conserved genomes. For this, we aligned the upstream region of *whiB5* orthologs from different pathogenic mycobacterial species strains and found that the two binding sites of Rv0023 are conserved across the analyzed mycobacterial species (Figure 6C). These results highlight the important residues for Rv0023 binding and its conservation across mycobacterial species.

### Rv0023 Binding Site Overlaps With the *whiB5* Promoter Region

**FIGURE 7:**
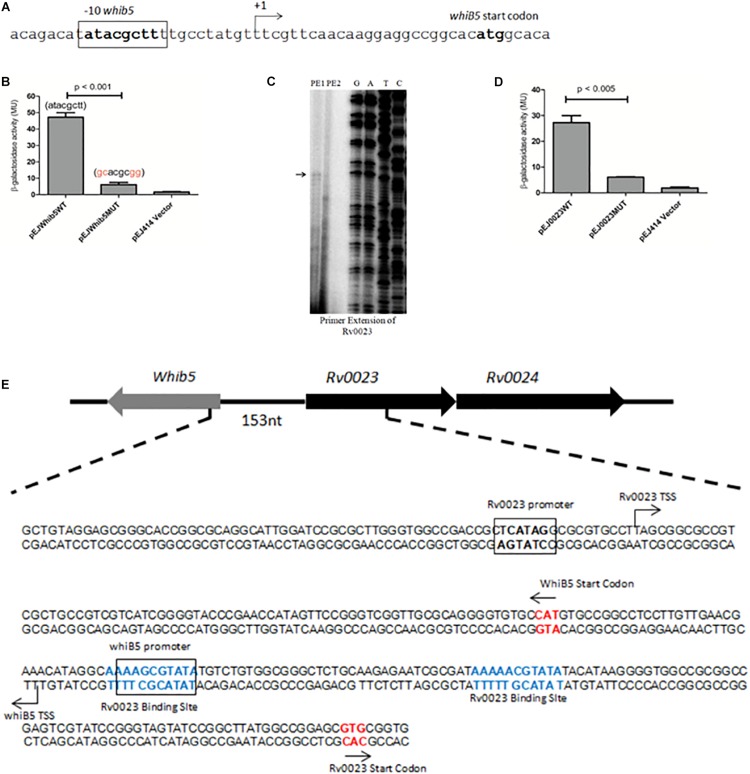
Identification of promoter regions of *whiB5* and *Rv0023*. **(A)** Nucleotide sequence of *whiB5* upstream containing *whiB5* start codon and –10 region. The –10 region is highlighted in bold. **(B)** β-Galactosidase activity of *Msmeg* transformed with *whiB5* wild-type promoter (pEJ*whiB5*WT), *whiB5* mutated (pEJ*whiB5*MUT) promoter, and vector control. **(C)** Primer extension analysis of *Rv0023*. PE1 and PE2 indicate two primers used for primer extension assay. The primer extension product is indicated by an arrow. G, A, T, and C indicate the sequencing ladder generated by using dideoxy nucleotides. **(D)** β-Galactosidase activity of predicted *Rv0023* wild type (pEJ0023WT) and *Rv0023* mutated (pEJ0023MUT) promoter showing significantly reduced activity of mutated promoter. **(E)** Genomic organization of Rv0023 binding sites (shown in bold and colored) and promoter regions (bold and boxed) of *whiB5* and *Rv0023*. Start codons are represented by an arrow.

In order to understand the mechanism of Rv0023-mediated repression of *whiB5*, the TSSs of *whiB5* and *Rv0023* were mapped. The putative −10 region of *whiB5* was identified earlier by RACE (Casonato et al., 2012) (Figure 7A). To test the functionality of the putative −10 region, we cloned the 250 bp upstream of *whiB5* into lacZ reporter vector (pEJ*whiB5*WT). The promoter activity was measured using the β-galactosidase assay in *Msmeg*. Mutations in the putative −10 region of *whiB5* (atacgctt to gcacgcgg) abolished the promoter activity, confirming the −10 region of *whiB5* (Figure 7B). To identify the promoter region of *Rv0023*, primer extension was performed. We identified the TSS at 228 bp upstream of *Rv0023* start codon (Figure 7C). A −10-like hexamer sequence TCATAG was identified upstream of TSS. To validate the functionality of the −10 region of *Rv0023*, 400 bp upstream of Rv0023 was cloned into a lacZ reporter vector (pEJ0023WT). β-Galactosidase assay with WT promoter and mutated promoter (pEJ0023MUT) indicated significant decrease in the promoter activity of the mutant strain compared to WT (Figure 7D). Mapping the promoters of *whiB5* and *Rv0023* with Rv0023 binding site revealed that Rv0023 binding site overlaps with the promoter of *whiB5* but not of the *Rv0023* promoter region (Figure 7E).

## Discussion

In this study, Rv0023, a member of the XRE family of transcriptional regulators, has been characterized, and its role in INH and ETH drug tolerance has been explored. The first part of our study concerns the role of Rv0023 in the physiology of mycobacteria. For our study, we used *Msmeg* as a surrogate model to study the effects of various stress conditions on wild type and Rv0023-expressing strains. It was observed that overexpression of Rv0023 confers higher tolerance toward INH and ETH in *Msmeg*. It was seen that the ectopic expression of Rv0023 alters the NADH/NAD^+^ ratio, thereby increasing drug tolerance. In the second part of the manuscript, we identified Rv0023 as a transcriptional regulator and studied the regulatory effect at *whiB5-Rv0023* locus and observed that Rv0023 negatively regulates *whiB5* expression but is not auto-regulatory. Rv0023 regulates *whiB5* expression by binding to specific sequences in the upstream region of *whiB5*. Our data indicate that there are two binding sites for Rv0023 upstream of *whiB5* and one of the sites overlaps with the *whiB5* promoter, thereby possibly occluding the binding of RNA polymerase. The binding site of Rv0023 is conserved across mycobacterial species as confirmed by multiple sequence alignment. Apart from this, we have also characterized the binding site of Rv0023 and found important bases for binding. While analyzing the spacer region, it was found that the length of the spacer region is important for proper binding.

Isoniazid, isonicotinic acid hydrazide, is a synthetic drug and the occurrence of INH-resistant strains is significantly more frequent than other drug-resistant *Mtb* clinical strains (Nachega and Chaisson, 2003). ETH, 2-ethylthioisonicotinamide, is a structural analog of INH and was first synthesized in 1956 (Grumbach et al., 1956). Many genes have been found to be associated with INH and ETH resistance in clinical isolates, but the role of transcription factors regulating INH and ETH co-resistance is poorly understood. Rv0023, a transcriptional regulator, conferring higher tolerance toward INH and ETH in *Msmeg*, provided us a chance to explore the role of transcription factors in INH and ETH co-resistance in *Mtb*. To elucidate the mechanism by which Rv0023 confers drug tolerance, we investigated two main genes known to be involved in INH and ETH co-resistance, *inhA* (enzyme involved in the synthesis of cell wall mycolic acid) (Banerjee et al., 1994) and *ndh* (NADH dehydrogenase maintains the NADH/NAD + ratio) (Miesel et al., 1998). We checked their expression levels in the vector control and Rv0023-expressing *Msmeg* strains and differential expression was only observed in the *ndh* gene. The *ndh* gene oxidizes NADH to NAD^+^, which maintains the ratio of NADH/NAD^+^ in mycobacterial cells (Jaworowski et al., 1981). Loss of *ndh* function leads to higher levels of NADH in the cytosol. Previous studies have shown that the higher NADH/NAD^+^ levels interfere with activation of INH/ETH drugs by competitive inhibition with the formation of INH-NAD and ETH-NAD and thus confer resistance to INH/ETH in *Msmeg* and BCG (Miesel et al., 1998; Vilcheze et al., 2005). So, we further measured the NADH/NAD^+^ ratio in vector control and Rv0023-expressing *Msmeg* strains. The NADH/NAD^+^ ratio was found to be increased in the Rv0023-overexpressed *Msmeg* strain, suggesting that negative regulation of *ndh* gene by Rv0023 is probably the mechanism by which Rv0023 confers INH and ETH tolerance in *Msmeg*. Our results were supported by previous studies where it was shown that Rv0023 regulon is enriched for NAD reductases (Rustad et al., 2014). Rv0023 did not bind to the upstream region of ndh, indicating that the regulation occurs possibly through indirect means, which may involve multiple intermediate gene products. Further studies are required to completely decipher this mechanism.

Synteny analysis has shown that *whiB5-Rv0023* locus is present mainly in pathogenic species of mycobacteria. The gene *whiB5* belongs to the WhiB family of transcriptional regulators, which are exclusive to actinomycetes, such as *Mycobacterium* and *Streptomyces* spp. (Soliveri et al., 2000). Earlier studies have shown that *whiB5* is a global transcriptional regulator, regulating 58 genes of diverse functions, including *sigM* and genes encoding for Type VII secretion systems such as *esx-2* and *esx-4*. It was shown that *whiB5* has a role in *Mtb* virulence and reactivation (Casonato et al., 2012). As the whole locus of *whiB5-Rv0023*-*Rv0024* is absent in *Msmeg*, it is difficult to gauge the true effect of Rv0023 in the physiology of *Mtb*. Further investigation in *Mtb* may reveal important aspects of Rv0023 regulation and its effects. Hence, taking into account the earlier studies and our current data, we surmise that Rv0023 plays a significant role in modulating genes associated with NADH/NAD^+^ levels, which further influences mycobacterial physiology.

In conclusion, our work shows, for the first time, that Rv0023 has a role in conferring INH and ETH tolerance in *Msmeg*. Understanding the role of transcriptional factors in the development of drug resistance will open new avenues in the field of drug discovery and may provide important insights into *Mtb* physiology.

## Data Availability Statement

The datasets generated for this study are available on request to the corresponding author.

## Author Contributions

AR, SG, RA, and SY conceived the hypothesis and rationale of the study, analyzed the results, and wrote the manuscript. SG performed all the experiments. CR contributed in cloning of constructs and recombinant protein purification.

## Conflict of Interest

The authors declare that the research was conducted in the absence of any commercial or financial relationships that could be construed as a potential conflict of interest.

## Footnotes

1 https://www.cdc.gov/tb/education/corecurr/

2 http://archaea.u-psud.fr/SyntTax
