# Supplementary material for: Ectopic Expression of Rv0023 Mediates Isoniazid/Ethionamide Tolerance via Altering NADH/NAD+ Levels in Mycobacterium smegmatis
Source: Front Microbiol. 2020 Feb 7;11:3. doi: 10.3389/fmicb.2020.00003 (PMC7020754; doi:10.3389/fmicb.2020.00003)
Supplement: Supplementary file 1 [file Table_1.docx]

**Supplementary Table 1 - Synteny analysis of *whiB5* and Rv0023**

| **Strain** | **Pathogenicity** | **whiB5-Rv0023 locus** |
| --- | --- | --- |
| *Mycobacterium bovis* | Pathogen | Present |
| *Mycobacterium africanum* | Pathogen | Present |
| *Mycobacterium canettii* | Pathogen | Present |
| *Mycobacterium tuberculosis* | Pathogen | Present |
| *Mycobacterium avium* | Pathogen | Present |
| *Mycobacterium marinum* | Pathogen | Present |
| *Mycobacterium kansasii* | Pathogen | Present |
| *Mycobacterium smegmatis* | Non -Pathogen | Absent |
| *Mycobacterium rhodesiae* | Non -Pathogen | Absent |
| *Mycobacterium gilvum* | Non -Pathogen | Absent |
| *Mycobacterium vanbaalenii* | Non -Pathogen | Absent |
