# Supplementary figures and images for: Ectopic Expression of Rv0023 Mediates Isoniazid/Ethionamide Tolerance via Altering NADH/NAD+ Levels in Mycobacterium smegmatis
Source: Front Microbiol. 2020 Feb 7;11:3. doi: 10.3389/fmicb.2020.00003 (PMC7020754; doi:10.3389/fmicb.2020.00003)

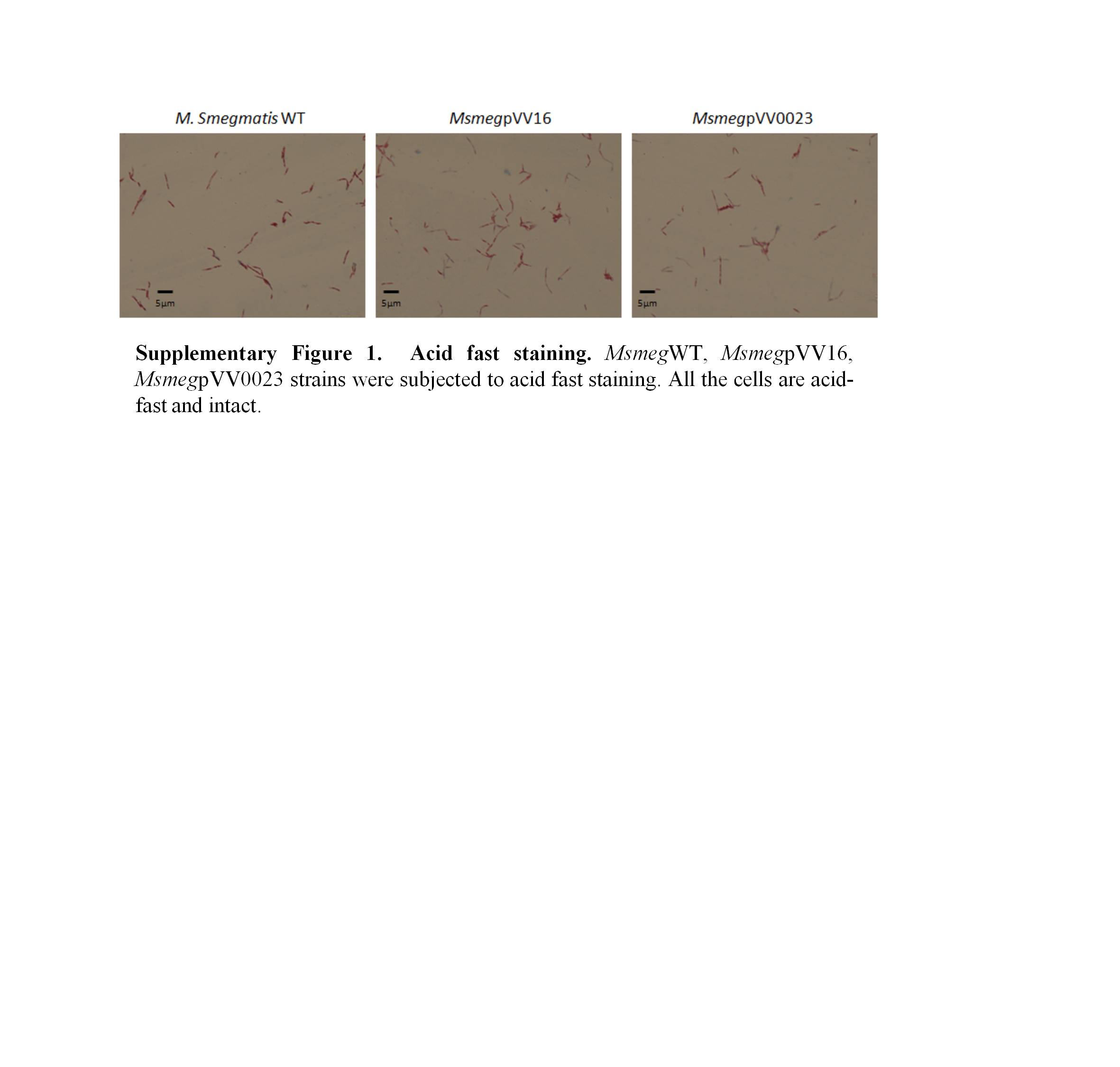

Supplement: Supplementary file 2 [file Image_1.jpg]

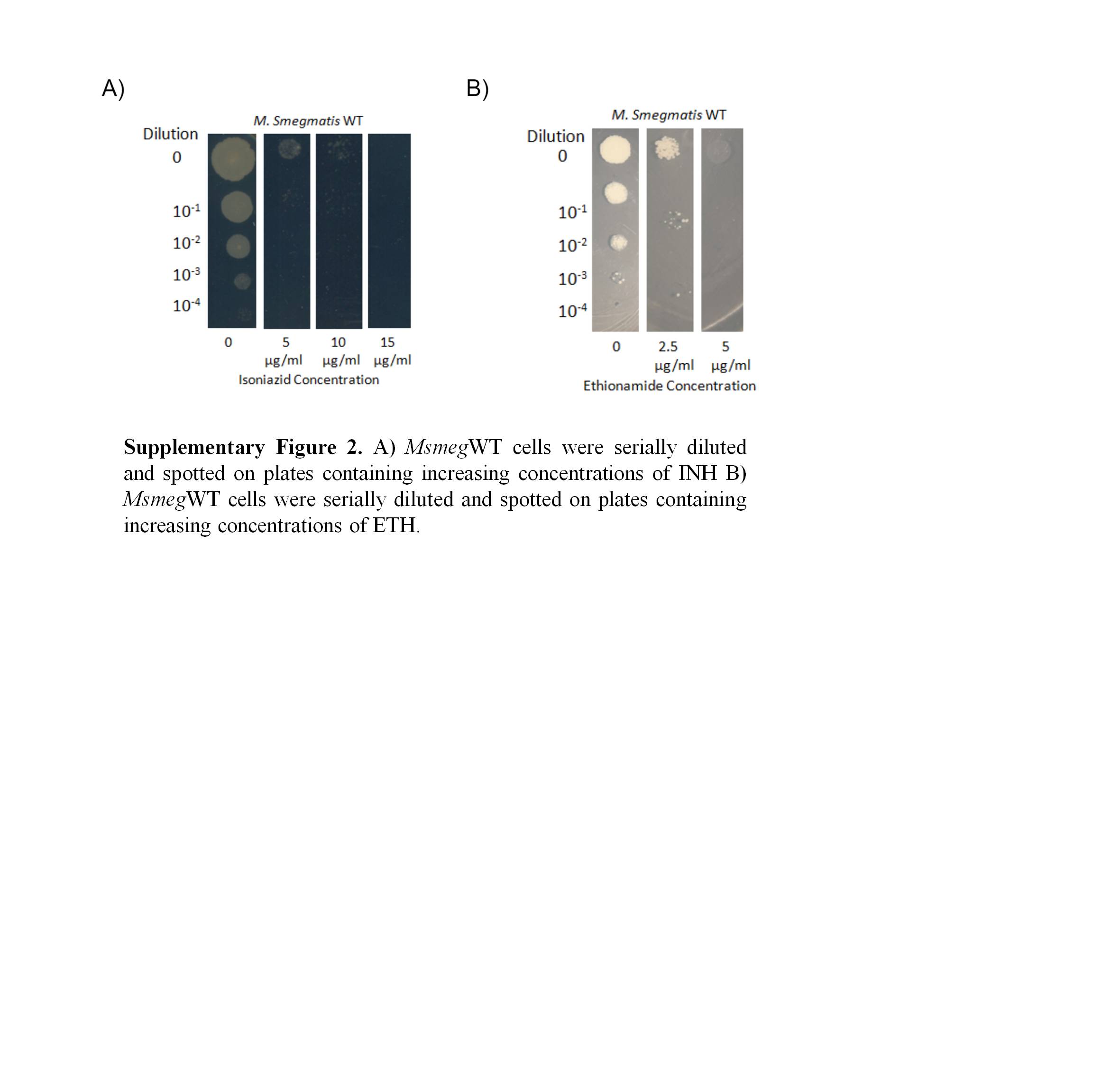

Supplement: Supplementary file 3 [file Image_2.jpg]

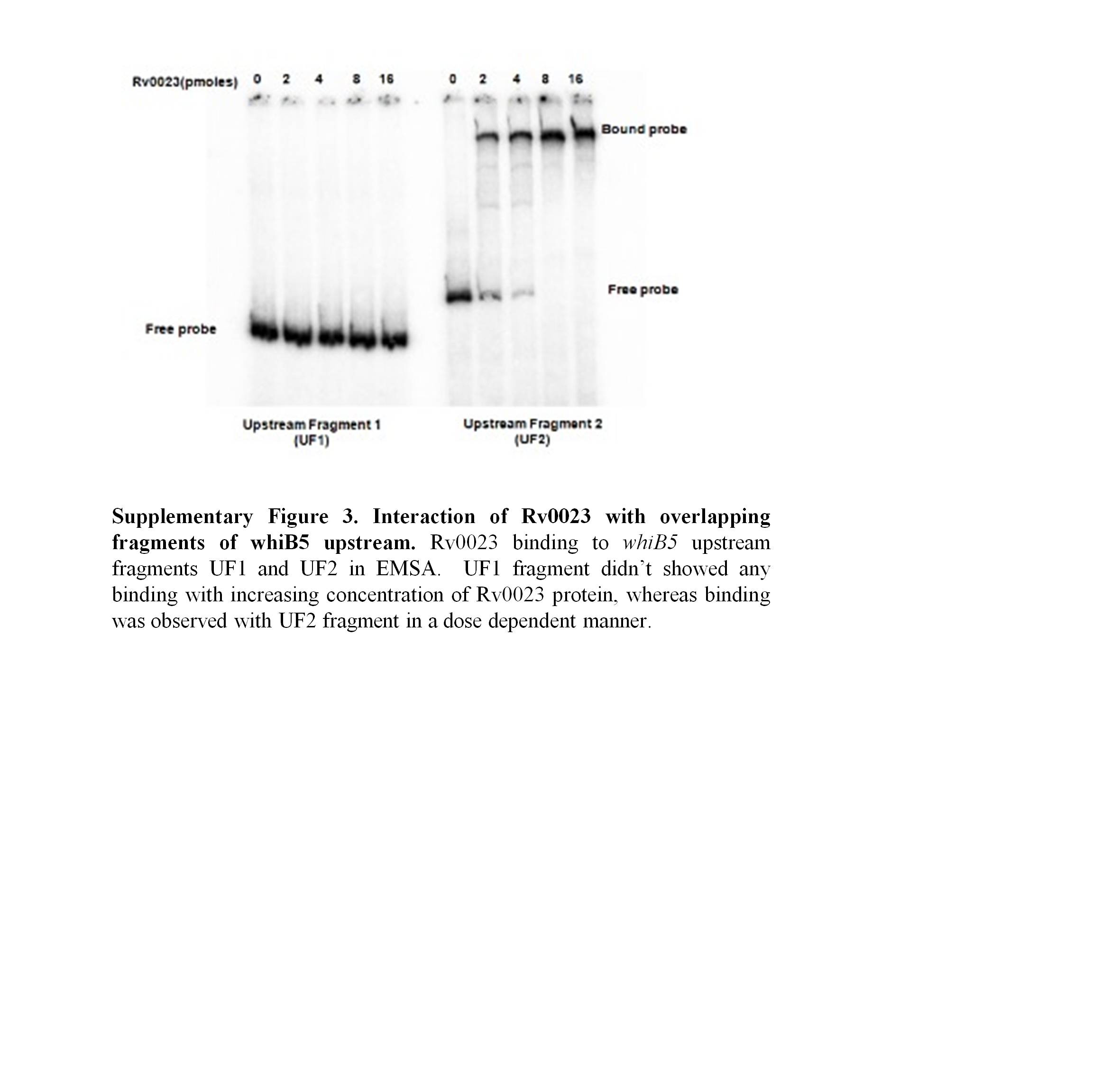

Supplement: Supplementary file 4 [file Image_3.jpg]
